# The *Trypanosoma cruzi* Protein TcHTE Is Critical for Heme Uptake

**DOI:** 10.1371/journal.pntd.0004359

**Published:** 2016-01-11

**Authors:** Marcelo L. Merli, Lucas Pagura, Josefina Hernández, María Julia Barisón, Elizabeth M. F. Pral, Ariel M. Silber, Julia A. Cricco

**Affiliations:** 1 Instituto de Biología Molecular y Celular de Rosario (IBR), Consejo Nacional de Investigaciones Científicas y Técnicas CONICET–Facultad de Ciencias Bioquímicas y Farmacéuticas, Universidad Nacional de Rosario, Rosario, Argentina; 2 Laboratory of Biochemistry of Tryps—LaBTryps -Departamento de Parasitologia, Instituto de Ciências Biomédicas, Universidade de São Paulo, Cidade Universitária, São Paulo, Brasil; 3 Área Biofísica, Departamento de Química Biológica, Facultad de Ciencias Bioquímicas y Farmacéuticas, Universidad Nacional de Rosario, Rosario, Argentina; Harvard School of Public Health, UNITED STATES

## Abstract

*Trypanosoma cruzi*, the etiological agent of Chagas' disease, presents nutritional requirements for several metabolites. It requires heme for the biosynthesis of several heme-proteins involved in essential metabolic pathways like mitochondrial cytochromes and respiratory complexes, as well as enzymes involved in the biosynthesis of sterols and unsaturated fatty acids. However, this parasite lacks a complete route for its synthesis. In view of these facts, *T*. *cruzi* has to incorporate heme from the environment during its life cycle. In other words, their hosts must supply the heme for heme-protein synthesis. Although the acquisition of heme is a fundamental issue for the parasite’s replication and survival, how this cofactor is imported and distributed is poorly understood. In this work, we used different fluorescent heme analogs to explore heme uptake along the different life-cycle stages of *T*. *cruzi*, showing that this parasite imports it during its replicative stages: the epimastigote in the insect vector and the intracellular amastigote in the mammalian host. Also, we identified and characterized a *T*. *cruzi* protein (TcHTE) with 55% of sequence similarity to LHR1 (protein involved in *L*. *amazonensis* heme transport), which is located in the flagellar pocket, where the transport of nutrients proceeds in trypanosomatids. We postulate TcHTE as a protein involved in improving the efficiency of the heme uptake or trafficking in *T*. *cruzi*.

## Author Summary

*Trypanosoma cruzi* is a protozoan parasite that causes Chagas’ disease, the most common parasitic disease in the Americas, but due to migration movements it has become relevant in non-endemic countries. *T*. *cruzi*, as other trypanosomatids responsible for human diseases (*T*. *brucei* and *Leishmania* spp.), lacks the enzymes for heme biosynthesis. However, it presents several heme-proteins involved in essential metabolic pathways, like biosynthesis of ergosterol and unsaturated fatty acids and mitochondrial cytochromes in its respiratory chain. In this work, we used different fluorescent compounds, which are structurally similar to heme (heme analogs), to explore the heme uptake in *T*. *cruzi*. The treatment of the different forms of *T*. *cruzi* with these compounds shows that this parasite is able to import heme during the replicative stages: epimastigote in the insect vector and intracellular amastigote in the mammalian host. We also characterized a *T*. *cruzi* protein, TcHTE, located in the flagellar pocket, where the transport of nutrients takes place in trypanosomatids. We propose TcHTE as a protein that improves the efficiency of the heme uptake. Considering the critical role of heme as an essential cofactor in *T*. *cruzi*, the identification of proteins involved in heme transport and distribution might result in the discovery of new molecular targets that may inhibit parasite proliferation.

## Introduction

*Trypanosoma cruzi* is the etiological agent of Chagas' disease or American trypanosomiasis. It is estimated that about 6 to 7 million people are infected worldwide, mostly in Latin America and southern states of USA, where Chagas’ disease is considered endemic. It is also becoming relevant in non-endemic regions due to migrations and the absence of control in blood banks and organ transplantation (http://www.who.int/mediacentre/factsheets/fs340/en/) [1,2]. *T*. *cruzi* has a complex life cycle alternating between two hosts and displaying at least four developmental stages. In the mammalian host, it is present as intracellular forms, mainly amastigotes (replicative stage) or transiently as intracellular epimastigotes, and as bloodstream trypomastigotes (non replicative, infective stage). In the insect vector, it is present as epimastigotes (replicative stage) and metacyclic trypomastigotes (non-replicative, infective stage). During the different life-cycle stages, the parasite faces different environments and has to adapt its metabolism to the nutritional availability through the different hosts [3,4].

*T*. *cruzi* presents nutritional requirements for several nutrients and cofactors, and heme is included in this list. As other trypanosomatids responsible for human diseases (*T*. *brucei* and *Leishmania* spp.), it lacks the enzymes for heme biosynthesis [5,6]. However these trypanosomatids present several heme-proteins involved in essential metabolic pathways like the biosynthesis of ergosterol, unsaturated fatty acids and mitochondrial cytochromes in the respiratory chain [7]. In this context, it is reasonable to hypothesize that *T*. *cruzi* must acquire heme from the extracellular environments along its life cycle.

Several mechanisms have been proposed for heme transport in different organisms. In gram-positive and gram-negative bacteria, direct heme uptake, bipartite heme receptors or hemophore-heme uptake systems were described [8,9]. In eukaryotic organisms some proteins were found playing a role as heme transporters, but none of them showed to be homologous to those of prokaryotes. The complete characterization of heme transport and distribution in these organisms is a long way from being completely understood yet [10,11]. As mentioned before, only a small number of proteins were identified and characterized as heme transporters. It is worth mentioning CeHRG-1 and CeHRG-4, both having a highly conserved function in modulating heme homeostasis in *Caenorhabditis elegans* [11–13]. CeHRG-4 was suggested to be involved in heme uptake by *C*. *elegans* intestinal cells and CeHRG-1 seems to mediate intracellular compartment heme delivery. Additionally, proteins involved in heme uptake have been identified in *Leishmania* spp. These proteins are the ABC transporter, ABCG5, responsible for intracellular heme traffic derived from the hemoglobin breakdown [14] and LHR1 postulated as a plasma membrane heme transporter [15]. Besides, it was shown that *T*. *cruzi* epimastigotes’ proliferation depends on heme in a dose dependent manner and that the heme uptake might occur *via* a specific porphyrin transporter, possibly a member of the ABC-transporter family [16,17]. In the present work, we show for the first time that heme transport takes place in the replicative life-cycle stages: the epimastigotes occurring in the midgut of the insect vector and the intracellular amastigotes occurring in mammalian host-cells. Notably, heme uptake does not occur in the infective non-replicative stage trypomastigotes. In addition, we characterize a *T*. *cruzi* protein (TcHTE), which promotes heme uptake when expressed in yeasts and improves the heme uptake/trafficking in *T*. *cruzi* epimastigotes. Remarkably, TcHTE is located in the flagellar pocket region, where it is postulated that nutrient transport takes place in trypanosomatids.

## Methods

### Reagents

Dulbecco's Modified Eagle Medium (DMEM) was obtained from Life Technologies. Fetal Bovine Serum (FBS) was purchased from Natocor SA (Córdoba, Argentina). δ-Aminolevulonic acid (δ-ALA), Hemin (Fe(III) protoporphyrin IX), Zn(II) mesoporphyrin IX (ZnMP), Zn(II) protoporphyrin IX (ZnPP), Sn(IV) mesoporphyrin IX (SnMP) and Ga(III) protoporphyrin IX (GaPP) were obtained from Frontier Scientific (Logan, UT, USA). Hemin was dissolved in 50% (v/v) EtOH, 0.01 M NaOH at a final concentration of 1 mM, filtered using a 22 μm syringe disposable filter and it was stored at -80°C. ZnMP, ZnPP, SnMP and GaPP were dissolved in DMSO at a final concentration of 10–20 mM and were stored at -80°C. The working solutions were prepared dissolving the stock solution in 0.01 M NaOH at a final concentration of 1 mM.

### Bacterial and yeast strains

*Escherichia coli* strains used for all cloning procedures were grown at 37°C in Luria–Bertani medium supplemented with ampicillin (100 μg/mL) or kanamycin (50 μg/mL) as necessary. The yeast *Saccharomyces cerevisiae* cells (*hem1*Δ) were grown either in a rich medium (YP, 1% yeast extract, 2% peptone), or in a synthetic complete medium (SC) lacking the appropriate nutrients for plasmid selection, supplemented with hemin or δ-ALA when it was necessary. In both cases, 2% Glucose (Glc) or 3% Glycerol–2% Ethanol (Gly–EtOH) were used as carbon sources. *S*. *cerevisiae hem1*Δ, *MATα*, *ura3-52*, *leu2-3*, *112*, *trp1-1*, *his3-11*, *ade6*, *can1-100(oc)*, *heme1*::*HIS3*, used for heterologous complementation assays, was generously provided by Dennis R. Winge, University of Utah. The yeast cells were transformed using the lithium acetate method [18].

### Cell lines and parasites

The Vero cell line (ATCC CCL-81, already available in our laboratory) was routinely maintained in DMEM medium supplemented with 0.15% (w/v) NaHCO_3_, 100 U/mL penicillin, 100 μg/mL streptomycin and 10% heat inactivated Fetal Bovine Serum (DMEM 10% FBS) at 37°C in a humid atmosphere containing 5% CO_2_. During the *T*. *cruzi* infections, Vero cells were incubated in DMEM medium supplemented with 0.15% (w/v) NaHCO_3_, 100 U/mL penicillin, 100 μg/mL streptomycin and 2% heat inactivated Fetal Bovine Serum (DMEM 2% FBS) at 37°C in a humid atmosphere containing 5% CO_2_. The epimastigotes of *T*. *cruzi* (Dm28c strain) were maintained in mid-log phase by successive dilutions through Liver Infusion Tryptose (LIT) medium supplemented with 10% heat inactivated FBS (LIT 10% FBS) and 20 μM hemin [19], at 28°C. Metacyclic trypomastigotes were obtained by spontaneous differentiation of Dm28c epimastigotes at 28°C. Cell-derived trypomastigotes were obtained after the infection of the monolayered Vero cells with metacyclic trypomastigotes. After two rounds of infections, the cell-derived trypomastigotes were used to perform analogs uptake assays or to infect Vero cells to obtain amastigotes. Intracellular amastigotes obtained 48–72 hours after the infection of the monolayered Vero cells were used for analogs uptake assay. The purity of all the obtained forms as well as their viability was evaluated by microscopic observation.

### Heme analogs uptake by the different *T*. *cruzi* life-cycle stages

#### Heme analogs transport assays in epimastigotes

The *T*. *cruzi* epimastigotes maintained in exponential growth phase in LIT 10% FBS supplemented with 20 μM hemin were collected by centrifugation, washed twice with PBS and 20 x 10^6^ cells were suspended in 100 μL of PBS. Later, 100 μL of 200 μM of the different HAs were added (the HAs final concentration was 100 μM) and incubated for 5 minutes at 28°C. The reaction was stopped by the addition of 800 μL of cold 0.5 mM hemin in PBS. Then, the cells were washed three times with cold PBS. Finally, the HAs uptake was detected by confocal microscopy (see below) or by fluorescence intensity (FI) measurement from total cell-free extracts (S1 Text). As a control, the viability of the treated epimastigotes was tested (S1 Text).

#### GaPP stability

To test the GaPP stability, epimastigotes maintained in exponential growth phase in LIT 10% FBS supplemented with 20 μM hemin (1 x 10^8^ cells) were collected by centrifugation washed with chilled 0.32 M sucrose and homogenized in pre-chilled buffer K (phosphate 90 mM pH 7.4 with 0.1% TritonX-100). Then, they were centrifuged (10 minutes at 600 x g) and the obtained clear supernatant was used to determine the GaPP degradation following the protocol used by Srivastava [20]. The reaction medium was complemented with 1.4 mM NADP^+^, 4 mM Glucose6P, 6.6 mM MgCl_2_, finally 2.5 μM GaPP was added and incubated at 37°C for 5 to 6 hours. The reaction was followed periodically up to 6 hours by recording the absorption spectra between 300 and 750 nm.

#### Heme analogs uptake by *T*. *cruzi* trypomastigotes

The cell-derived trypomastigotes (as described above) were collected by centrifugation of the supernatant of the cell culture at 4000 x g at room temperature for 10 minutes. Then, they were washed twice with PBS, suspended in 60 μL of PBS and incubated for 30 minutes with 60 μL of 200 μM of the different HAs (100 μM of HAs final concentration). The HAs transport was stopped by the addition of 300 μL of formaldehyde 3.7% (w/v) in PBS. Then the cells were washed twice with PBS and suspended in 40 μL of PBS. HAs uptake was evaluated by confocal microscopy.

#### Heme analogs uptake by intracellular *T*. *cruzi* amastigotes

First, the HAs transport and toxicity were examined in the Vero cell line. To evaluate the HAs toxicity, Vero cells previously plated on 96 multi-well plate in DMEM 2% FBS and incubated for 24 hours at 37°C in a humid atmosphere containing 5% CO_2_, were washed twice with PBS and incubated with 200 μL of DMEM 2% FBS supplemented with 5, 20, 50 or 100 μM of the different HAs for 30 minutes or 2 hours. The viability of cells was measured by MTT dye (3-{4,5-Dimethylthiazol-2-yl}-2,5-diphenyltratazolium Bromide, Sigma-Aldrich) colorimetric method. DMEM 2% FBS was used as control of 100% of viability. Data is expressed as means ± SD of the results of three independent replicas of each assayed condition. The HAs uptake was evaluated by confocal microscopy at 30 minutes and 2 hours of incubation. To obtain the intracellular amastigotes, the Vero cell line was plated on cover glasses in DMEM 2% FBS at 37°C in a humid atmosphere containing 5% CO_2_. After 24 hours, cells were infected with trypomastigotes of *T*. *cruzi* Dm28c in a proportion of 10 trypomastigotes/cell. Four hours after infection, the cells were washed twice with PBS and 2 mL of DMEM 2% FBS was added. The cells were cultured at 37°C, in a humid atmosphere containing 5% CO_2_. 48 hours post-infection (when we observed intracellular amastigotes by microscopy), the cells were washed twice with PBS, then 200 μL of DMEM 2% FBS supplemented with 100 μM of the different HAs was added and incubated for 30 minutes. After that, the cells were washed tree times with PBS and fixed with methanol for 15 minutes. Again, they were washed twice with PBS and mounted with VectaShield reagent (Vector Laboratories). The HAs uptake was evaluated by confocal microscopy. As a control, non-infected cells were treated with HAs and then analyzed likewise the infected cells.

### *In silico* analysis

The orthologous sequences of *LHR1* in other trypanosomatids were obtained from TriTrypDB (http://tritrypdb.org/tritrypdb/) [24] using LmjF.24.2230 from *Leishmania major* as seed. Amino acids multiple sequence alignments were carried out using the version 2.0 of Clustal W and Clustal X software (http://www.ebi.ac.uk/Tools/msa/clustalw2, [25]). Transmembrane-domains were predicted with TMHMM software, accessible from ExPASy Bionformatics Resource Portal (http://www.cbs.dtu.dk/services/TMHMM-2.0, [26]).

### Cloning of *T*. *cruzi TcHTE* coding sequence and analysis of it expression in yeast cells

#### Cloning the *T*. *cruzi TcHTE* gene

The *T*. *cruzi* coding sequence of *TcHTE* was amplified by PCR using genomic DNA from Dm28c epimastigote as template. The primers listed below were designed to amplify the gene introducing the *Bam*HI restriction sites at the 5´-end and *Xho*I restriction sites with a 6xHis epitope tag at the 3´-end, generating the *TcHTE*.*6HIS* insert.

FP.TcHTE: 5´-GC**GGATCC**ATGGCTATTTCAGAAAAGAAGTG-3´; RP1.TcHTE: 5’-CCG**CTCGAG**TTAATGATGATGATGATGATGACCCATAATATCTGCGTTCTTTTCG-3’.

The *TcHTE*.*6HIS-GFP* construct was made cloning the following two PCR products. First, the *TcHTE*.*6HIS* sequence was amplified by PCR, using FP.TcHTE and RP2.TcHTE (5´-CGC**CTCGAG**ACCATGGTGATGGTGATGATG-3´) primers to eliminate the STOP codon. Second, the GFP sequence was amplified using the following primers from a plasmid available in the laboratory. These primers introduced the *Xho*I restriction site at the 5´-end and *Eco*RI-*Eco*RV-*Xba*I at the 3´-end; FP.GFP: 5´-TAC**CTCGAG**ATGAGCAAGGGCGAGGAGCTG-3´; RP.GFP: 5´-CC**TCTAGATATCGAATTC**TTCACGACTTGTACAGCTCGTCC-3´. The first PCR product was treated with *Bam*HI and *Xho*I, and the second product was treated with *Xho*I and *Xba*I restriction enzymes. Both fragments were cloned together to form the *TcHTE*.*6HIS-GFP* product. Later, the *TcHTE*.*6HIS* and *TcHTE*.*6HIS-GFP* products were cloned into a pENTR3C vector (Gateway system, Invitrogen) and their sequences solved. Then, the inserts were transferred by recombination using LR clonase II enzyme mix (Invitrogen) to *T*. *cruzi* expression vector p*Tc*INDEX-GW [27]—an integrative vector derivate of p*Tc*INDEX [28] containing an inducible expression site under tetracycline control. For yeast assays, the same inserts were cloned in the p426.M25 inducible vector under the control of the MET25 promoter [29], previously used in our laboratory [30].

#### Spot growth assay

*S*. *cerevisiae hem1*Δ transformed with p426.MET25 as control or with the *TcHTE*.*6HIS* and *TcHTE*.*6HIS-GFP* containing plasmids, were grown in SC medium supplemented with 250 μM δ-ALA at 30°C ON with shaking. Then, the cells were harvested by centrifugation at 4000 x g for 15 minutes and the pellets were washed with water. The yeast cells were suspended at a final OD of 2 and four serial dilutions were performed. 7 μL of each dilution were plated in solid SC medium supplemented with Glucose or Glycerol-Ethanol as carbon source and with different hemin concentration or δ-ALA as control. The cells were incubated at 30°C for 4–5 days.

#### Immunoblotting

The total protein extracts from *S*. *cerevisiae* expressing *TcHTE*.*6HIS-GFP* were separated by electrophoresis on a 12% SDS polyacrylamide gel and electrotransferred onto nitrocellulose membranes (Amersham). The membranes were blocked in 5% non-fat dry milk in PBS supplemented with 0.05% Tween 20 (PBS-T) for 1 h. Followed by the detection with rabbit anti-GFP antibody (1:1000) (Santa Cruz Biotechnology) or mouse anti-His antibody (1:3000) (Calbiochem). After washing with PBS-T, the membranes were incubated with peroxidase-labeled anti-rabbit IgG (Sigma) diluted 1:15000 in PBS-T or peroxidase-labeled anti-mouse IgG (Sigma), diluted 1:15000 in PBS-T. Bound antibodies were detected with ECL Prime Western Blotting Detection kit (GE Healthceare). When it was necessary, the membranes were stripped 30 minutes at 50°C with Stripping Buffer (2% SDS, 100 mM β-mercaptoethanol, 62.5 mM Tris pH 8).

### Analysis of *T*. *cruzi* epimastigotes expressing *TcHTE*.*6HIS* or *TcHTE*.*6HIS-GFP* genes

#### Parasites transfection

*T*. *cruzi* Dm28c epimastigotes harboring the pLEW13 plasmid, Dm28c.pLEW13 [28], were grown in LIT 10% FBS supplemented with 20 μM hemin and 250 μg/mL G418 to approximately 3 × 10^7^ cells/mL. Parasites were then harvested by centrifugation at 4000 × g for 5 minutes at room temperature, washed twice with PBS and suspended to a cell density of 1 × 10^8^ cells/mL in 0.4 mL of electroporation buffer pH 7.5 (137 mM NaCl, 2.7 mM KCl, 4.7 mM Na_2_HPO_4_, 1.47 mM KH_2_PO_4_, 0.5 mM MgCl_2_, 0.1 mM CaCl_2_). The cells were transferred to a 0.2 cm gap cuvette and 15 to 30 μg of DNA was added. The mixture was placed on ice for 10 minutes and then subjected to 1 pulse of 450 V and 500 μF using GenePulser II (Bio-Rad, Hercules, USA). After electroporation, cells were maintained on ice until being transferred into 2 mL of LIT 10% FBS supplemented with 20 μM hemin, and then were incubated at 28°C. After 24 h, the antibiotic (hygromycin B) was added to an initial concentration of 50 μg/mL. Then, 72 to 96 hours after electroporation, cultures were diluted 1:10 and concentration of antibiotic was doubled. Stable resistant cells were obtained approximately 30 days after transfection.

#### The effect of heme on epimastigotes growth of *T*. *cruzi* expressing *TcHTE*.*6HIS-GFP*

The epimastigotes transfected with p*Tc*INDEX-GW (control) or the *TcHTE*.*6HIS-GFP* cloned in p*Tc*INDEX-GW were maintained in LIT 10% FBS (without hemin) for 48 hours, then they were challenged to growth on 2.0 mL LIT 10% FBS without (0 μM) or supplemented with 5 and 20 μM hemin, and 0.5 μg/mL of tetracycline was added for gene expression induction. The transfectant parasites were maintained in mid-log phase by periodic dilutions every 2 days. The cell growth was monitored by cell counting in a Neubauer chamber.

Epimastigotes containing the *TcHTE*.*6HIS-GFP* cloned in p*Tc*INDEX-GW vector were maintained in LIT 10% FBS with 5 μM hemin and 0.5 μg/mL of tetracycline for 3 days. Then, they were washed twice with PBS and fixed with 3.7% (w/v) formaldehyde in PBS. Cells were settled on polylysine-coated coverslips, mounted with the VectaShield reagent, and used for confocal microscopy.

#### Flagellar complex isolation

To isolate the flagellar complex we followed the protocol proposed by Sasse and Gull with minor modifications [31]. Epimastigotes expressing *TcHTE*.*6HIS-GFP* were collected, washed with PBS and settled on polylysine-coated coverslips. The cells were incubated in MME buffer (10 mM MOPS, pH 6.9, 1 mM EGTA, 1 mM MgSO_4_) containing 0.1% Triton X-100 on ice for 10 minutes, the buffer was changed three times. Then the cytoskeleton were incubated in MME buffer containing 1 M NaCl and 0.1% Triton X-100 on ice with one buffer change, until the subpellicular microtubules were judged by phase-contrast microscopy to be solubilized. The flagellar complexes were washed with PBS, fixed with 3.7% (w/v) formaldehyde in PBS for 20 minutes and cold methanol for 5 minutes. Then, the flagellar complexes were washed with PBS and permeabilized with 0.2% Triton X-100 in PBS for 5 minutes. After that, they were washed again with PBS and incubated with monoclonal mouse antibody anti-trypanosome α tubulin clone TAT-1 (a gift from K. Gull, University of Oxford, England, UK) diluted in UIB (150 mM NaCl, 150 mM Tris pH 7.5, 1% BSA) for 2 hours at room temperature. Non-bound antibodies were washed with 0.1% Tween 20 in UIB and then incubated with rabbit anti-mouse IgG (Alexa Fluor 555, Molecular Probes) for 1 hour. The slides were washed twice with 0.1% Tween 20 in UIB and once with PBS. Finally the slides were mounted with the Vecta-Shield reagent and analyzed by confocal microscopy.

### Confocal microscopy

The epimastigotes were fixed with 500 μL of formaldehyde 3.7% (w/v) in PBS. Then they were washed twice with PBS, suspended in 40 μL of PBS, settled on polylysine-coated coverslips and mounted with VectaShield reagent. Also the trypomastigotes incubated with HAs were settled on polylysine-coated coverslips and mounted with VectaShield reagent. Additionally, the infected and non-infected Vero cells treated with HAs were mounted with VectaShield reagent. All the images were acquired with a confocal Nikon Eclipse TE-2000-E2 microscope containing the following fluorescent filter cubes: 99748-C55027 and 99749-C87049, and using Nikon EZ-C1 software. The emission of the samples displaying red fluorescence (treated with HAs or treated with secondary antibody conjugated with Alexa Fluor 555) was registered at 605/75nm. The emission of the samples containing GFP fusion proteins was registered at 515/30 nm. The emission of the samples treated with DAPI was registered at 450/30 nm. All the images were processed using the ImageJ software [21,22].

### Heme content analysis

The heme content was quantified by the alkaline pyridine method [23]. 50–250 x 10^6^ cells of *T*. *cruzi* epimastigotes were collected and washed twice with PBS. Samples were suspended in 500 μL with PBS and then combined in a 1 mL cuvette with 500 μL of a stock solution containing 0.2 N NaOH and 40% (v/v) pyridine. The oxidized and reduced (by addition of sodium dithionite) spectra were recorded between 450 nm and 700 nm. The 557 nm peak was identified in the differential -reduced minus oxidized- spectrum. The heme concentration was estimated using the molar extinction coefficient 23.98 mM^−1^ cm^−1^.

### Statistical analysis

The experiments were performed by triplicate, and the data is presented as the mean ± SD (standard deviation). All the assays were independently reproduced at least 2–4 times. Statistically significant differences between groups (p values less than 0.05) were assessed using Mann Whitney U test (intracellular heme content) or Kruskal-Wallis one-way analysis of variance by ranks followed by Dunn's multiple comparison post-test (HAs cytotoxicity effect on Vero cells), as appropriate (GraphPad, version 3.0).

## Results

### Heme analogs were transported by *T*. *cruzi* replicative life-cycle stages

**Fig 1:**
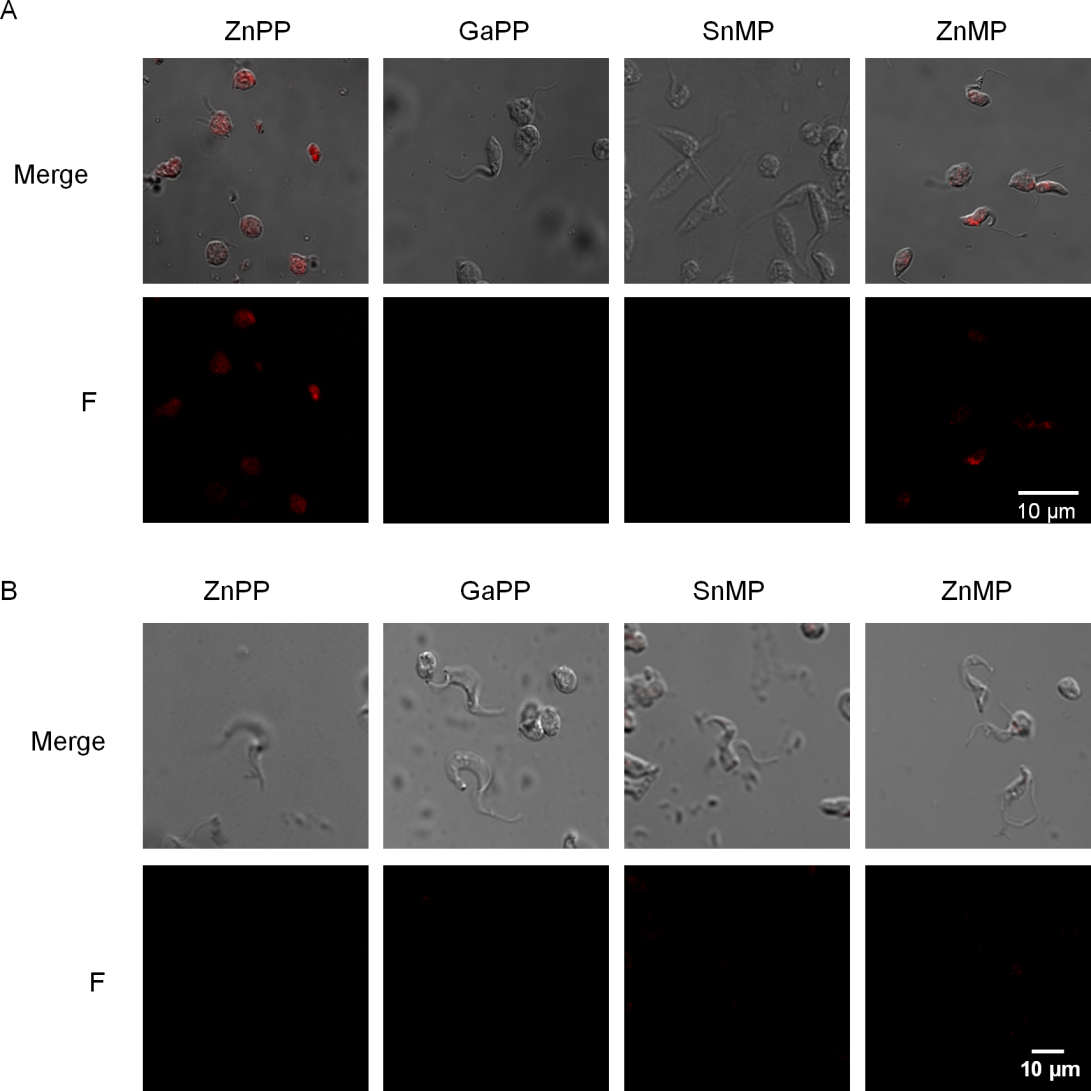
Heme analogs are selectively imported by *T*. *cruzi* epimastigotes but HAs are not imported by trypomastigotes. Heme analog uptake was evaluated by confocal microscopy images from (A) epimastigotes incubated for 5 minutes and (B) trypomastigotes incubated for 30 minutes, at 28°C with 100 μM of HAs. The results are representative of at least three independent experiments.

As previously reported, the 10 minutes treatment with heme analogs (SnPP, PdMP and ZnMP) prevented heme incorporation by epimastigotes and competed with heme for its transport [17]. Therefore, it is rational to use this type of compounds to trace the heme transport in other life-cycle stages than epimastigotes. In this work we used the derivatives of protoporphyrin IX ZnPP (Zn(II) protoporphyrin IX) and GaPP (Ga(III) protoporphyrin IX) and the derivatives of mesoporphyrin IX ZnMP (Zn(II) mesoporphyrin IX) and SnMP (Sn(IV) mesoporphyrin IX) to trace heme transport along the different *T*. *cruzi* life-cycle stages. All these heme analogs (HAs) are fluorescent and mimic heme in structure (S1 Fig). First, we confirmed HAs transport in the epimastigote stage, where exponential growth phase epimastigotes were incubated with the different HAs (100 μM in PBS) for 5 minutes and HAs incorporation was analyzed by confocal microscopy (Fig 1A). The confocal images revealed that ZnPP and ZnMP were clearly imported and maintained by the parasite while samples treated with GaPP and SnMP did not show the expected signal. To confirm this observation a fraction of treated epimastigotes were lysed and the soluble fraction (total cell-free extracts) was analyzed by direct FI measurements (S1 Text and S2 Fig). Parasites treated with ZnPP, ZnMP and less extent with GaPP displayed a fluorescent signal, while those treated with SnMP did not show any (Figs 1A and S2A). The viability of the treated epimastigotes was also evaluated, therefore aliquots of treated parasites were taken, washed and incubated in LIT 10% FSB plus hemin, and the cell growth was followed up for four days. We did not detect irreversible toxicity as consequence of the 5 minutes treatment with these HAs (S2B Fig).

As mentioned above, the fluorescent signal of the epimastigotes incubated with GaPP was weaker than those of ZnPP and ZnMP (S2 Fig) and was not detected by confocal microscopy (Fig 1A). This fact could be due to three possibilities: 1) the analog could have been degraded, 2) the analog could have been exported back to the extracellular medium or 3) the GaPP transport was less efficient than the other HAs transport. To determine the possible GaPP degradation by any enzymatic activity (like heme oxygenase activity), we evaluated the stability of this compound in presence of total cell-free extract of epimastigotes. The analog specific absorbance was followed up during 5 hours and no significant change on the acquired spectra was observed, ruling out the degradation of GaPP by intracellular components. Alternatively, it was possible to postulate that the observed low FI signal could be the result of an inefficient import process or a rapid export response, determining a stationary state with low intracellular concentrations of GaPP. On the other hand, it remains to be elucidated why SnMP is not accumulated into the cell at detectable level as other HAs.

**Fig 2:**
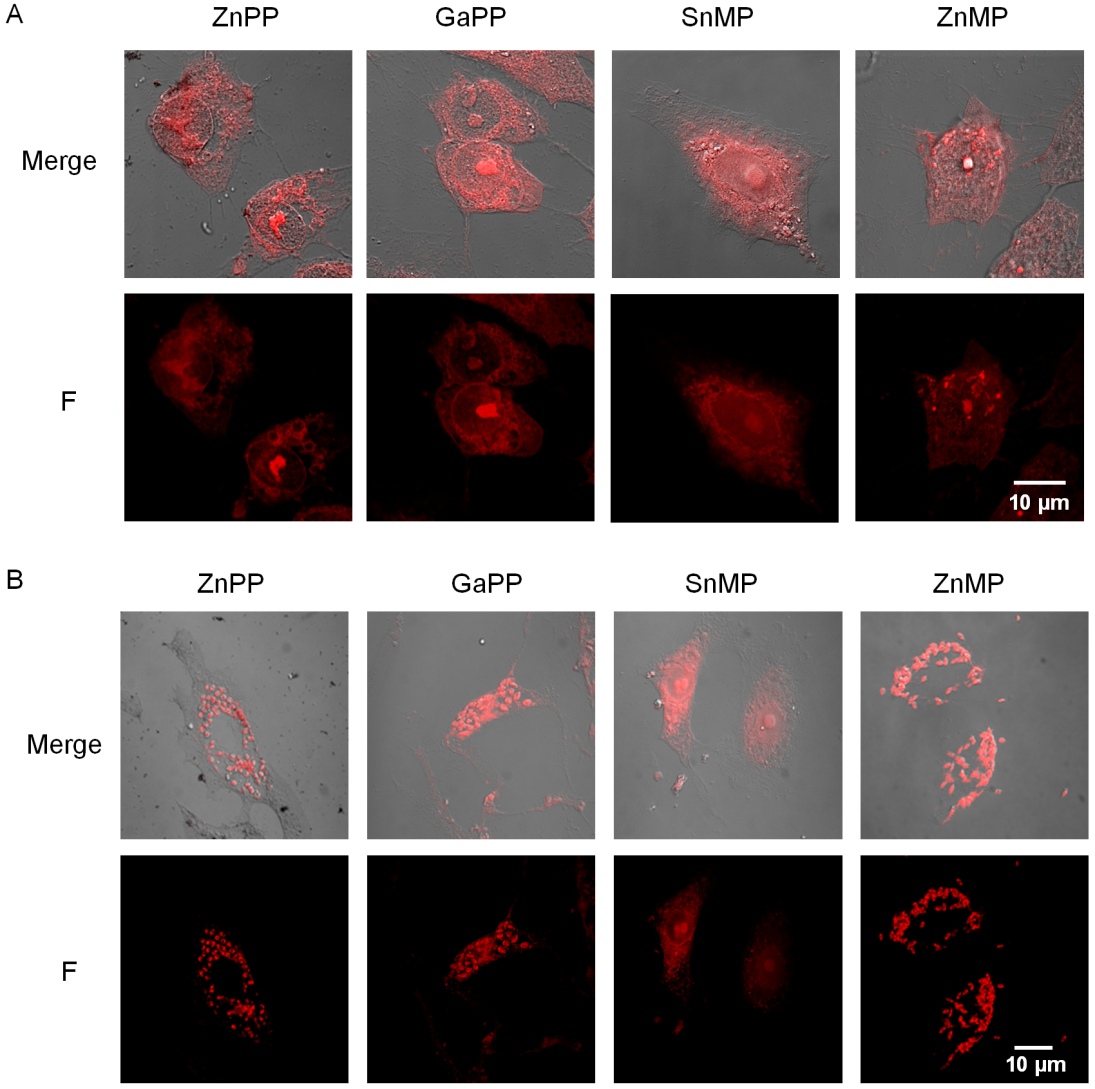
Heme analogs are transported by amastigotes. Confocal microscopy images of uninfected Vero cells (A) and infected Vero cells with amastigotes of *T*. *cruzi* (B) incubated with 100 μM heme analogs for 30 minutes. The results are representative of at least three independent experiments.

To explore if *T*. *cruzi* is able to import heme in other life-cycle stages, we evaluated the transport of these compounds by trypomastigotes using similar assays to those used for epimastigotes. In this case, cell-derived trypomastigotes were incubated 30 minutes with 100 μM of HAs and then analyzed by confocal microscopy. Surprisingly, a fluorescent signal was not observed in samples treated with any of the used HAs (Fig 1B), suggesting that trypomastigotes did not import heme, at least under these experimental conditions. We were also interested in evaluating the heme transport in the intracellular *T*. *cruzi* amastigotes, the replicative life-cycle stage during the mammalian host infection. To accomplish this, we first evaluated the toxicity and transport of the HAs into the mammalian host cells. The Vero cells were treated with 100 μM of the analogs (ZnPP, GaPP, ZnMP and SnMP) and after 2 hours their viability and the HAs uptake were evaluated. No toxicity was detected in these experimental conditions (S3 Fig), and 30 minutes of incubation were enough to allow the compounds to get inside and become available for the amastigotes (Fig 2A). After that, non-infected and infected cells (2–3 days post infection) were treated with 100 μM analogs for 30 minutes and analyzed by confocal microscopy (Fig 2A and 2B). All HAs–including SnMP- produced a homogeneously distributed fluorescent signal inside the non-infected cells, showing that they were imported and maintained in the intracellular environment. However, when infected Vero cells were incubated with HAs, a completely different fluorescent pattern was observed (Fig 2B). After the treatment with ZnPP, ZnMP and GaPP, they showed a punctuated fluorescent signal overlapping with the location of intracellular amastigotes. Interestingly, infected cells treated with SnMP showed the same fluorescent pattern as non-infected cells. These results strongly suggest that the HAs are being taken up by the cells and, except for SnMP, efficiently imported by *T*. *cruzi* amastigotes. Remarkably, intracellular amastigotes could discriminate between the assayed HAs, as previously observed for epimastigotes.

### TcHTE protein promoted heme transport in *hem1*Δ yeast cells

After searching in *T*. *cruzi* databases, we identified a gene (AYLP01000162 from Dm28c genomic sequence and TcCLB.504037.10 and TcCLB.511071.190 from CL Brener genomic sequence) that encodes for a protein with a 55% sequence similarity to LHR1, a heme transporter identified in *L*. *amazonensis* [15] and 38% with CeHRG-4 (*C*. *elegans* protein [12]). The *T*. *cruzi* protein sequence was scanned *in silico* for transmembrane spanners in order to predict its secondary structure and our results indicated the presence of four probable transmembrane domains (TMD) as it was also predicted for LHR1 and CeHRG-4. Besides their similarities–in sequence and predicted topology- two essential tyrosine residues present in LHR1 (Y18 and Y129 [32]) are conserved in the *T*. *cruzi* protein, which correspond to Y16 and Y127 following the *T*. *cruzi* protein numbering. However, another relevant tyrosine (Y80 in LHR1) is substituted by a phenylalanine, F78, in the *T*. *cruzi* protein. Nevertheless, none of these residues are conserved in the *C*. *elegans* protein CeHRG-4 (S4 Fig). In the same way, other residues or motifs relevant for CeHRG-4 activity were not conserved in the *T*. *cruzi* protein. This evidence pointed out that the *T*. *cruzi* putative protein could play a role in *T*. *cruzi* heme transport therefore we decided to clone its gene for further characterization.

**Fig 3:**
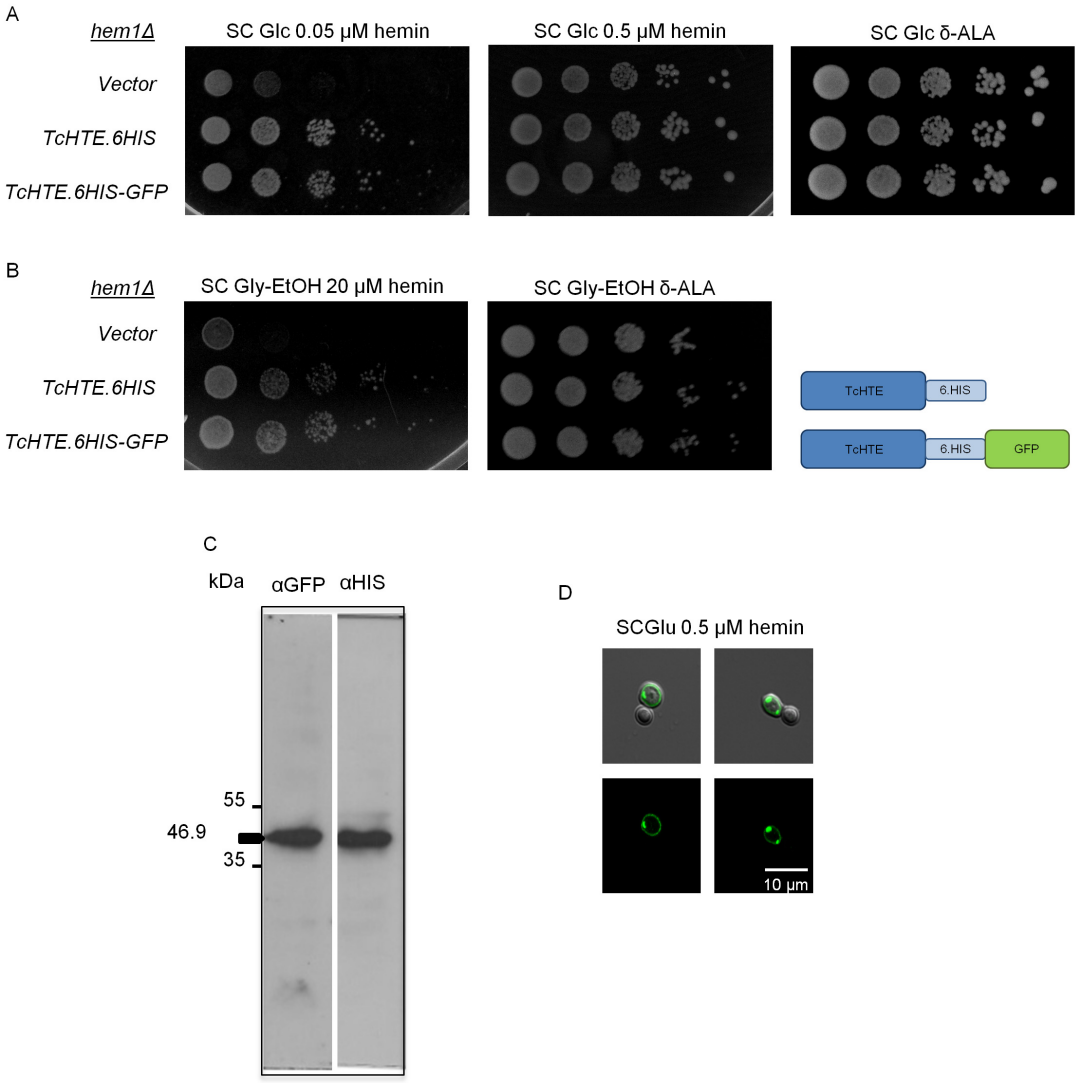
TcHTE promotes heme uptake in *hem1*Δ yeast cells. Spot growth assay of *S*. *cerevisiae hem1*Δ transformed with the p426.MET25 or the vector carrying *TcHTE*.*6HIS* or *TcHTE*.*6HIS-GFP* constructs. 7 μL of four serial dilutions from the initial cultures of OD = 2 were plated in a solid SC medium with Glucose (A) or Glycerol-Ethanol (B) as a carbon source, and different hemin concentrations, or δ-ALA as control. Western blot of total cell extracts of *S*. *cerevisiae* transformed with vector carrying *TcHTE*.*6HIS-GFP* with anti-GFP or anti-His antibodies (C). Confocal microscopy images of *S*. *cerevisiae hem1*Δ transformed with a vector carrying *TcHTE*.*6HIS-GFP* growth in SC medium with Glucose and 0.5 μM hemin (D).

The complete ORF was amplified, sequenced and cloned upstream of a sequence encoding for a C-terminal His-tag or a His-GFP-tag. To find the possible participation of this *T*. *cruzi* putative protein in heme transport we tested its activity in *hem1*Δ yeasts (deficient in heme synthesis, [33]). Both constructs were cloned into a yeast expression vector, which were used to transform *hem1*Δ cells. The transformed yeasts were exposed to a selective medium supplemented with different hemin concentrations. The yeast cells expressing the *T*. *cruzi* gene (both versions) were able to grow at a lower hemin concentration compared to the control (Fig 3), indicating that, similarly to LHR1 [15,32], the *T*. *cruzi* protein promoted heme transport by *hem1*Δ yeast cells (Fig 3A) and also allowed the recovery of the respiratory growth when a non-fermentable carbon source such as glycerol-ethanol was used (Fig 3B). The presence of the *T*. *cruzi* protein (6His-GFP version) in total cell extracts was verified by western blot analysis (Fig 3C). Taking into account that heme uptake has been improved by the expression of the *T*. *cruzi* gene, we named its product TcHTE for *T*. *cruzi* Heme Transport Enhancer protein.

The yeast cells expressing a functional TcHTE.6His-GFP were used to analyze the protein location by confocal microscopy (Fig 3D). The images confirmed that TcHTE.6His-GFP is present in the plasma membrane, as it was expected for proteins involved in transport processes.

### TcHTE.6His-GFP expression negatively affected *T*. *cruzi* epimastigote growth

**Fig 4:**
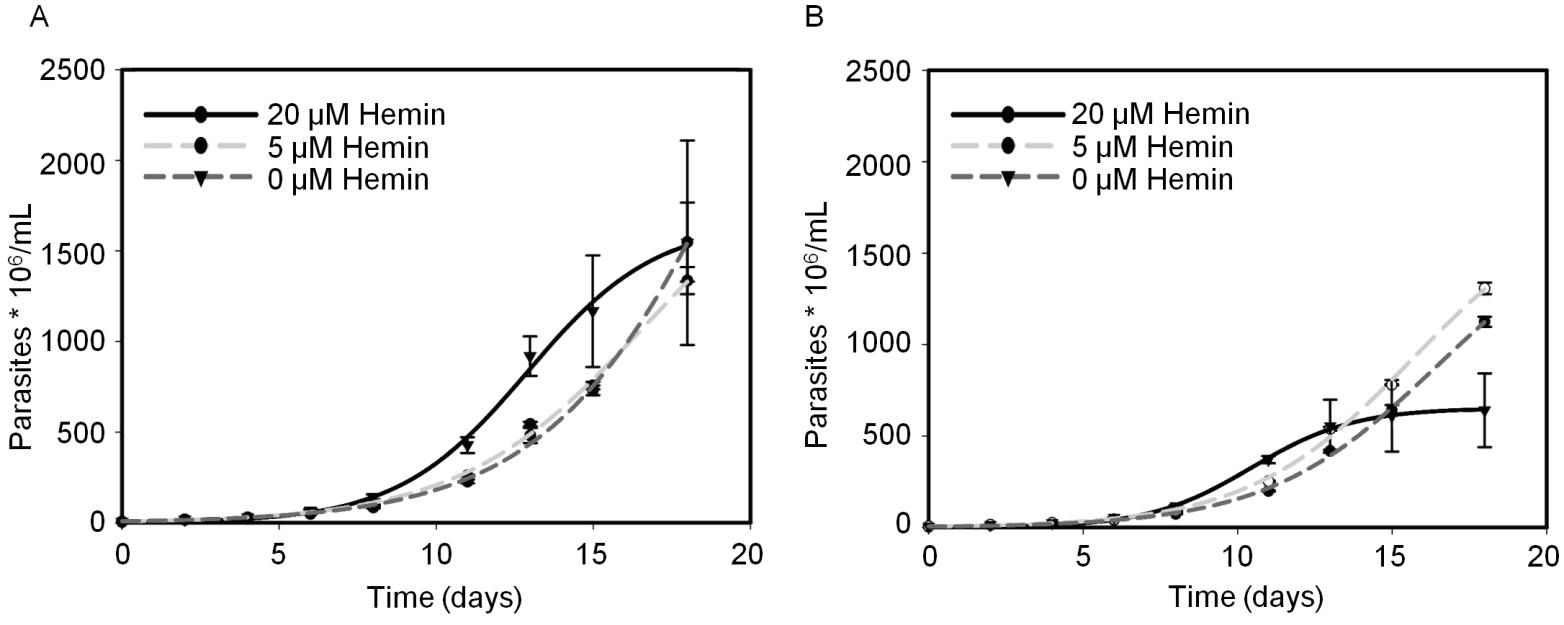
TcHTE.6His-GFP negatively affects epimastigote growth under standard heme concentrations. *T*. *cruzi* epimastigote (Dm28c.pLEW13) transfected with the p*Tc*INDEX-GW vector (A) or the *TcHTE*.*6HIS-GFP* cloned in p*Tc*INDEX-GW vector (B) were maintained in LIT 10% FBS, supplemented with 0, 5 or 20 μM hemin with periodic dilutions every 2 days. The results are representative of at least two independent experiments. The experimental data is presented as the mean ± SD.

Considering that the addition of 15–30 μM hemin is an essential requirement to maintain epimastigotes of *T*. *cruzi* in all axenic media used up to now, first we evaluated the parasite’s growth when the medium was supplemented with different concentrations of hemin. We did not observe differences in its growth (S5 Fig) or in the intracellular heme content when the epimastigotes were maintained with 5 or 20 μM hemin (3.572 ± 0.012 and 3.667 ± 0.129 nmol heme/10^9^ parasites respectively, p>0.05). After that, to unveil the role of TcHTE in *T*. *cruzi*, we analyzed the epimastigotes’ growth and heme content in parasites over-expressing the *TcHTE* gene. In this case, Dm28c.pLEW13 epimastigotes were transfected with the *TcHTE6HIS-GFP* versions of the gene cloned in the inducible *T*. *cruzi* expression vector p*Tc*INDEX-GW or with the empty vector as control. After selection, the parasites containing the *TcHTE*.*6HIS-GFP* gene or the empty vector were used to test their growth under different conditions of heme availability. First, these transfected parasites were kept for 72 hours in the absence of hemin, and then the cultures were either maintained without the addition (0 μM hemin) or supplemented with 5 and 20 μM hemin. In all cases 0.5 μg/mL of tetracycline was added to induce the gene expression and the growth was monitored. We did not observe significant differences when these parasites were maintained in a medium supplemented with 5 μM or without hemin, suggesting that the induction of *TcHTE*.*6HIS-GFP* gene expression did not alter the parasite growth in these conditions (Fig 4A and 4B). Surprisingly, when parasite cultures were supplemented with 20 μM hemin, the growth was moderately impaired in epimastigotes expressing the *TcHTE*.*6HIS-GFP* gene compared to the control as it is shown in Fig 4B. Also the intracellular heme content was quantified in epimastigotes maintained in 20 μM hemin, and it significantly increased from 3.1 ± 0.2 in the control to 5.3 ± 0.5 nmol heme/10^9^ parasites in the epimastigotes expressing the *TcHTE*.*6HIS-GFP* gene (p<0.05).

### TcHTE.6His-GFP was located in the flagellar pocket region of epimastigotes

**Fig 5:**
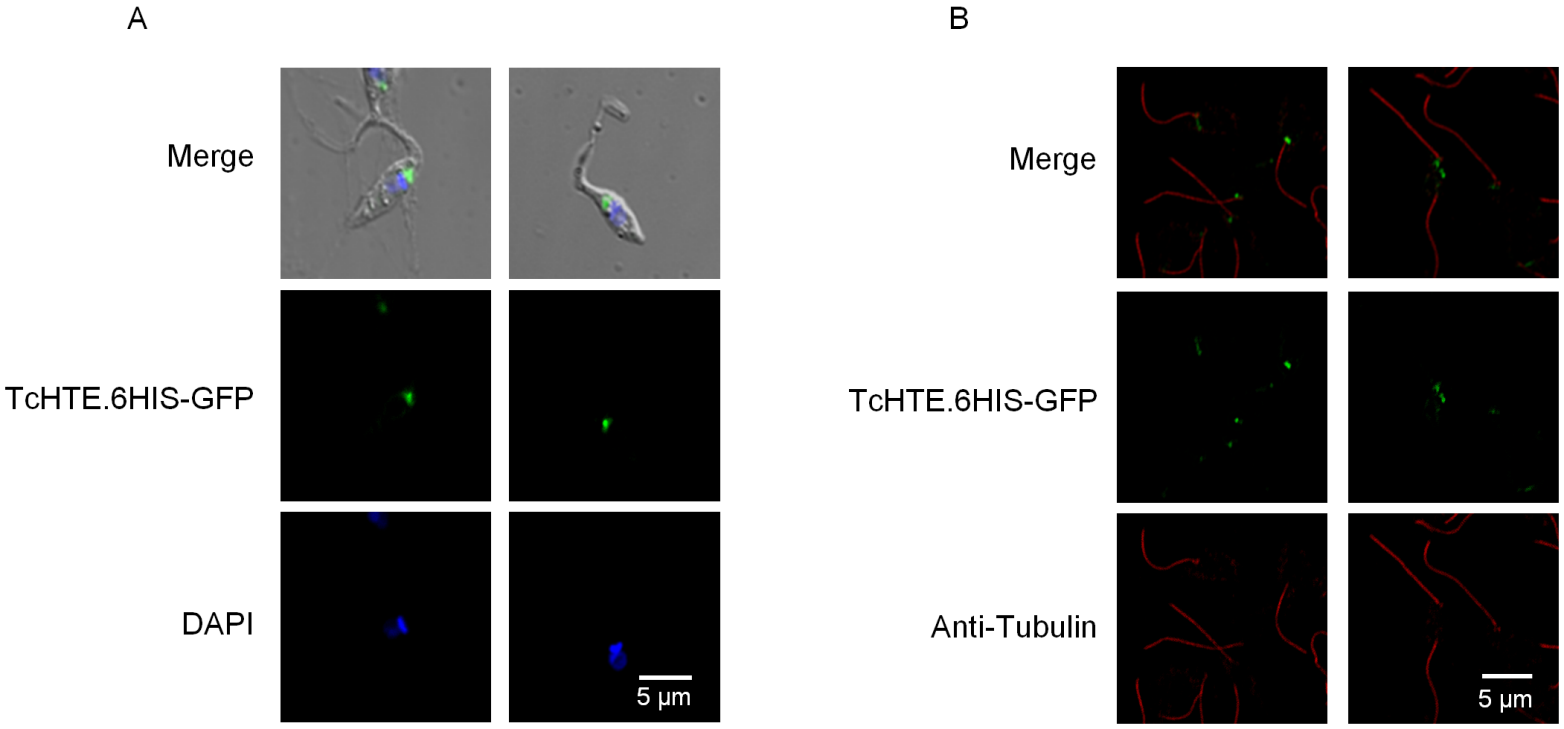
TcHTE.6His-GFP locates in flagellar pocket. Confocal microscopy images of the epimastigotes expressing *TcHTE*.*6HIS-GFP* (A), and isolated flagellar complexes of epimastigotes expressing TcHTE.6His-GFP (green) treated with anti-tubulin antibodies (red) (B).

Finally, we evaluated the location of the TcHTE.6His-GFP protein. In that case, transfected parasites expressing *TcHTE*.*6HIS-GFP* were analyzed by confocal microscopy. Most of GFP fluorescent signals were punctually located in a region corresponding to the flagellar pocket close to the flagellum emerging site, where it is postulated that metabolite transport processes take place in the *T*. *cruzi* (Fig 5A). The TcHTE.6His-GFP location was verified by the flagellar complex isolation assay where the GFP signal was observed next to the flagellar complex (Fig 5B).

## Discussion

*Trypanosoma cruzi* requires a supply of heme like other trypanosomatids, which are auxotrophic for this cofactor. In view of these facts, it is postulated that *T*. *cruzi* must import it from the different hosts along its different life-cycle stages [7]. Since heme is a toxic molecule, it is accepted that these organisms must possess an essential and controlled pathway for its uptake and distribution [6,34]. Recently, several proteins were described as heme transporters or involved in heme transport in trypanosomatids [14,15] and other organisms with heme auxotrophy like *C*. *elegans* [35]. Among them, CeHRG-4 and CeHRG-1 proteins from *C*. *elegans* [12,13] and LHR1 from *L*. *amazonensis*, were described as proteins involved in heme transport or playing a role as a heme transporter. Also, LHR1 activity was relevant for the infectivity of *L*. *amazonensis* [32,36]. In addition, the activity of an ABC transporter–ABCG5- was reported as a heme transporter involved in the intracellular heme distribution in *L*. *donovani* [14]. Moreover, it was demonstrated that heme is relevant for *T*. *cruzi* epimastigote proliferation and that its uptake was inhibited by the treatment with ABC transporters’ inhibitors, indicating that heme transport should be dependent on an active transporter [16,17]. Nevertheless, up to date, the identity of the *T*. *cruzi* heme transporter was not unraveled.

In this work we used different fluorescent heme analogs (ZnPP, GaPP, ZnMP and SnMP) to study heme transport along the different *T*. *cruzi* life-cycle stages. Only ZnPP, ZnMP, and to a lesser extent GaPP (only detected by direct fluorescent measurements) were accumulated inside the epimastigotes. The weak fluorescent signals detected in parasites incubated with GaPP could be a consequence of its slower uptake, its faster export, its degradation, or any combination of them. Since we were not able to detect GaPP degradation in the presence of total cell-free extracts we exclude this possibility. This reinforces the idea that the reduced intracellular concentration of GaPP might be a consequence of an unfavorable relationship between influx and efflux when compared to ZnMP and ZnPP. On the other hand, SnMP was not accumulated at detectable levels in epimastigotes. These results suggest that the HAs were selectively taken up by the *T*. *cruzi* epimastigotes.

In spite of its relevance, no information was found, up to now, about heme transport in other life-cycle stages besides epimastigotes. Our results reveal that only replicative stages (epimastigotes and amastigotes), but not infective stages (trypomastigotes), were able to uptake heme. Moreover, based on our observations, we postulate that *T*. *cruzi* amastigotes are able to selectively withdraw most of the HAs (ZnPP, ZnMP and GaPP) available from their environment, the host cell cytosol. Apparently, SnMP did not accumulate in intracellular amastigotes as it was observed in epimastigotes. These results reinforce the hypothesis that *T*. *cruzi* presents an exclusive heme transporter that might discriminate between structurally related compounds. Remarkably, the heme uptake regulation during the life cycle together with the proposed selectivity of the heme transporter points that this process is critical, being very specific and tightly regulated.

Recently, LHR1 was identified as a protein relevant for heme transport in *L*. *amazonensis* [32]. Therefore, we described a *T*. *cruzi* protein -herein named TcHTE- that shows sequence similarity to LHR1 and also with the *C*. *elegans* protein CeHRG-4 [12]. In spite of their sequence similarity and the conserved predicted topology, TcHTE, as well as LHR1 and CeHRG-4, does not show any recognized domains or motifs that allow its classification as an active or ABC transporter [37,38], as it was expected based on previous reported results [17]. Nevertheless, the hypothetical *T*. *cruzi* protein was characterized. TcHTE activity was first evaluated in the yeast *hem1*Δ (*HEM1* gene encodes for the 5-aminolevulenic acid synthase, the first enzyme in heme biosynthesis) as it was previously used to assess the heme transport activity of CeHRG-4 and LHR1 proteins [13,15]. Since yeast exhibit a low affinity transport activity, *hem1*Δ cells can grow in a medium supplemented with high concentrations of hemin. The expression of the *TcHTE* gene enhanced heme transport and/or distribution in these yeast cells allowing their growth at lower hemin concentrations and also the recovery of the respiratory growth. Moreover, TcHTE was located on the yeast's plasma membrane as it was expected for a transporter protein, the same was observed with LHR1 [14,35] and Ce-HRG4 [13]. Nevertheless the results presented here do not allow us to discriminate if TcHTE is itself the heme transporter or if it is an essential protein that promotes/enhances the heme transport (or trafficking) activity.

Finally, we explored the role of TcHTE in *T*. *cruzi* epimastigotes. We did not observe significant differences when epimastigotes overexpressing the *TcHTE* gene were grown in a medium supplemented with low hemin concentrations. However, when these epimastigotes were exposed to 20 μM hemin–the standard hemin concentration used in axenic medium- the presence of extra TcHTE turned out to be a disadvantage to maintain the optimal growth and an increment in the intracellular heme was observed. In the later case, the extra TcHTE could push on the heme uptake rendering more intracellular heme than the parasite is able to metabolize causing a mild negative effect on growth rates. These observations strongly suggest that TcHTE displays a role enhancing heme transport or trafficking. Additionally, epimastigotes transfected with a plasmid containing *TcHTE*.*6HIS-GFP* were analyzed by confocal microscopy. The GFP fluorescent signal was located on the flagellar pocket region, similarly to the reported hemoglobin receptor of *Leishmania donovani* [39]. Surprisingly, TcHTE was not homogenously distributed along the plasma membrane or in internal vesicles as it was observed for LHR1 on *L*. *amazonensis* promastigotes [15]. These observations might indicate differences in heme transport between these trypanosomatids, which could be mediated by different transporters and/or involving different mechanisms. Although many transporters have been biochemically characterized in *T*. *cruzi*, their location remains unknown [4,40,41]; however it is established that the trypanosomatids’ transport proceeds through the flagellar pocket [39,42]. Two different mechanisms (or pathways) were proposed for heme acquisition by *T*. *cruzi* epimastigotes. One of them, probably *via* the endocytosis, is responsible for the hemoglobin import, and the other, a faster pathway (few seconds), allows the transport of free heme (followed by the fluorescent heme analog PdMP) depending on an active transporter [16]. In both cases the fluorescent signal (from the heme analog PdMP or hemoglobin-rhodamine) was initially observed in the anterior part of the parasite (on the flagellar pocket region), which corresponds with the cellular location for TcHTE protein. Although the TcHTE protein itself does not exhibit any distinctive signature allowing its classification as an active transporter, the results presented here allowed us to postulate that TcHTE plays a relevant role in promoting/enhancing the heme transport in order to regulate this activity. However the identity of the heme transporter still remains unknown.

In summary, the results presented here show, for the first time, that *T*. *cruzi* transports heme along its replicative life-cycle stages, the epimastigote in the midgut of the insect vector and the intracellular amastigote in the mammalian host, but not in the infective form, the trypomastigote stage. Our findings revealed the existence of a protein transporter that might discriminate between structurally related compounds. Besides, we presented the first characterization of a *T*. *cruzi* protein, named TcHTE, which is conserved in trypanosomatids. We postulate that TcHTE plays a relevant role in *T*. *cruzi* heme transport/trafficking enhancement regulating this activity, without excluding it as part of or associated with the transporter complex. Considering the critical role of heme as an essential cofactor in *T*. *cruzi*, and the fact that its uptake inhibition results deleterious for the parasite, new approaches will be required for the complete identification of the molecular partners involved in its transport and distribution. Their identification and characterization might result in new molecular targets to inhibit parasite proliferation.

## Supporting Information

S1 Fig Heme analogs structure, and their absorption and fluorescence spectra. (A) Molecular structure of the derivatives of protoporphyrin IX: Ga(III) protoporphyrin IX (GaPP), Zn(II) protoporphyrin IX (ZnPP) and Fe(III) protoporphyrin IX (hemin), and derivates of mesoporphyrin IX: Sn(IV) mesoporphyrin IX (SnMP) and Zn(II) mesoporphyrin IX (ZnMP). (B) Absorbance spectra. (C) Fluorescence spectra of the analogs excited at 405 nm.
(TIF)

S2 Fig HAs uptake by *T*. *cruzi* epimastigotes followed by fluorescence measurements and the control of HAs treatment toxicity. Heme analog uptake was evaluated by FI measurements from epimastigotes incubated for 5 minutes at 28°C with 100 μM of HAs (A). λex = 405 nm and λem = 583 nm for GaPP, 578 nm for ZnMP, 588 nm for ZnPP and 574 nm for SnMP. As control of treatment toxicity, the samples incubated for 5 minutes with these HAs were washed with PBS and suspended in LIT 10% FBS supplemented with 20 μM hemin. The cells were then maintained without periodic dilutions for 4 days and growth was monitored by cell counting in a Neubauer chamber (B). The results are representative of at least three independent experiments. The experimental data is presented as the mean ± SD.
(TIF)

S3 Fig Heme analogs cytotoxicity evaluated in Vero cells. Vero cells were incubated for 2 hours at 37°C in a humid atmosphere containing 5% CO_2,_ with different dilutions of the heme analogs and the cytotoxicity was measured with MTT viability assay. The results are representative of at least three independent experiments. The experimental data is presented as the mean ± SD.
(TIF)

S4 Fig Sequence alignment of TcHTE, LHR1 and CeHRG-4 proteins. The alignment was performed using the Clustal W software [25], available at the EMBL-EBI website. Transmembrane-domains, represented by light frames, were predicted with TMHMM software [26], accessible from ExPASy Bionformatics Resource Portal. The reported conserved histidine residues of CeHRG-4 and LHR1 (residues H108 and H105 respectively) are outlined in black squares and according to these analyses, are absent in TcHTE. The Y18 reported as essential in LHR1 is conserved in TcHTE as well as Y129 (Y16, and Y127 in TcHTE) outlined in red, but Y80 which is not present, was replaced for F78 in TcHTE, outlined in yellow.
(TIF)

S5 Fig The growth of *T*. *cruzi* epimastigotes monitored at different hemin concentrations. *T*. *cruzi* epimastigote maintained in LIT 10% FBS supplemented with 20 μM hemin in exponential growth phase were collected by centrifugation, washed twice with PBS and suspended at 4.5 x 10^6^ cells per 1.5 mL of LIT 10% FBS supplemented with 0, 2, 5, 20, 50 or 100 μM hemin. The cultures were maintained in the mid-log phase by periodic dilutions every 2 days and the growth was monitored by cell counting in a Neubauer chamber. The experimental data is presented as the mean ± SD. The results are representative of at least three independent experiments.
(TIF)

S1 Text Direct fluorescence intensity measurements from cell-free extracts of *T*. *cruzi* epimastigotes. (DOCX)
