# Supplementary figures and images for: The Trypanosoma cruzi Protein TcHTE Is Critical for Heme Uptake
Source: PLoS Negl Trop Dis. 2016 Jan 11;10(1):e0004359. doi: 10.1371/journal.pntd.0004359 (PMC4713871; doi:10.1371/journal.pntd.0004359)

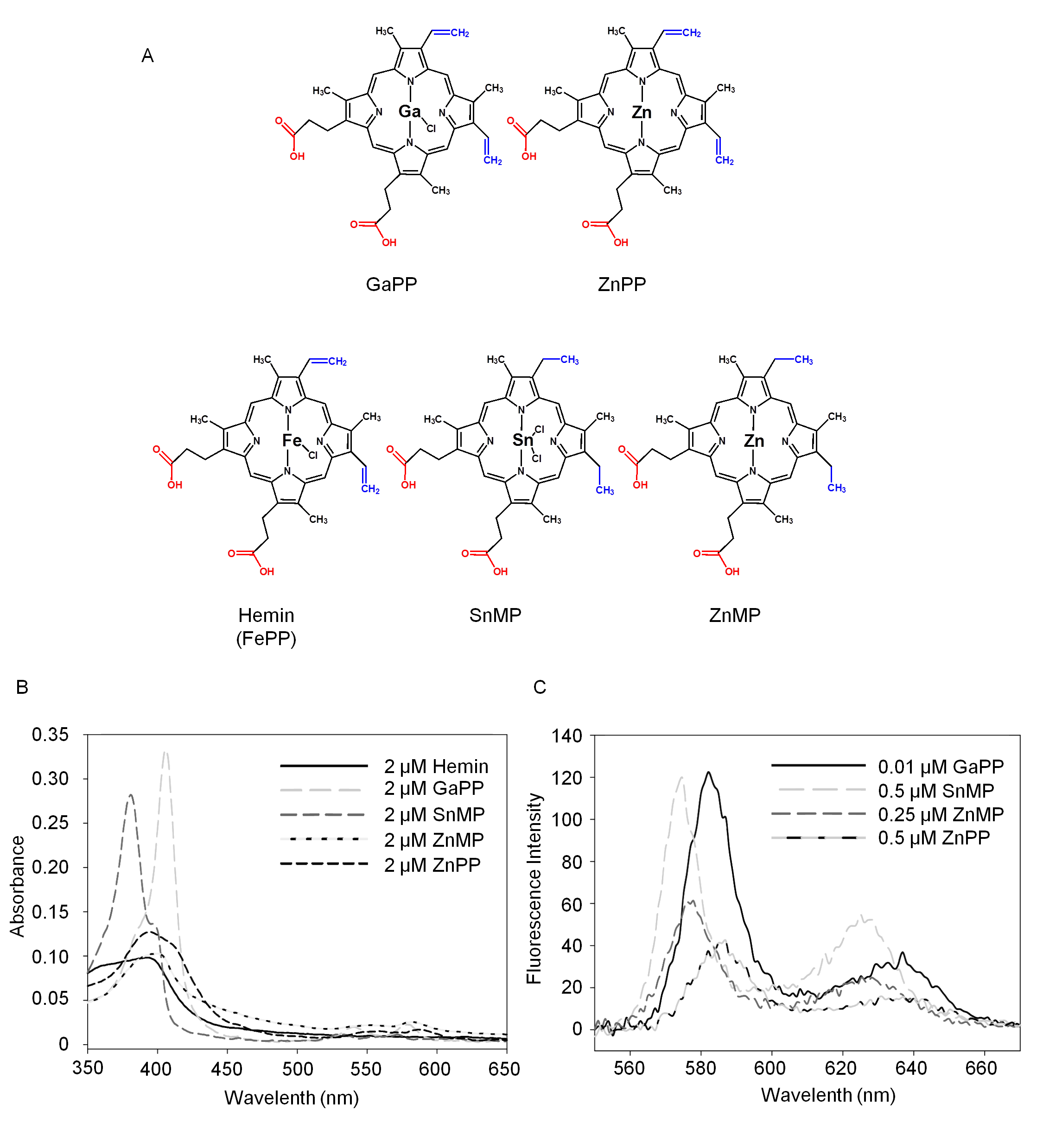

Supplement: S1 Fig — (A) Molecular structure of the derivatives of protoporphyrin IX: Ga(III) protoporphyrin IX (GaPP), Zn(II) protoporphyrin IX (ZnPP) and Fe(III) protoporphyrin IX (hemin), and derivates of mesoporphyrin IX: Sn(IV) mesoporphyrin IX (SnMP) and Zn(II) mesoporphyrin IX (ZnMP). (B) Absorbance spectra. (C) Fluorescence spectra of the analogs excited at 405 nm. (TIF) [file pntd.0004359.s001.TIF]

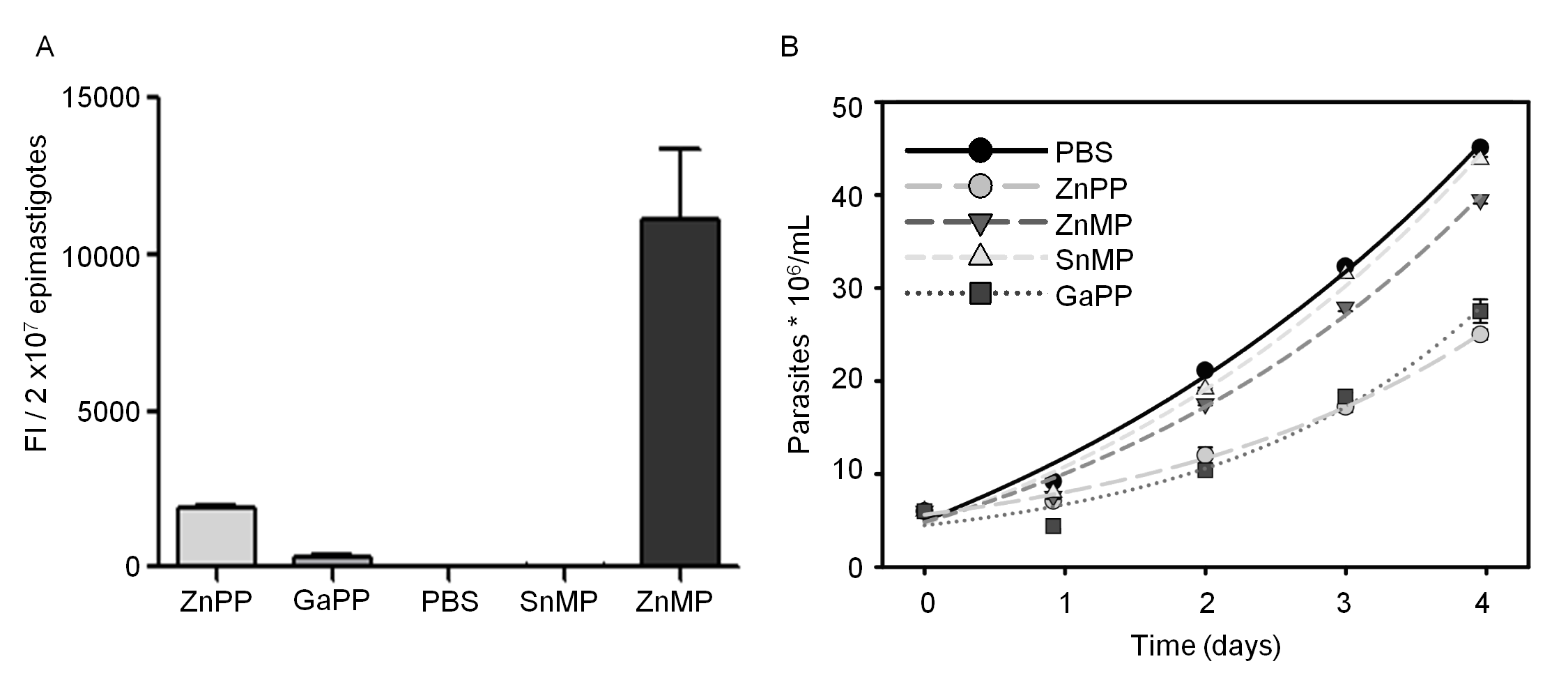

Supplement: S2 Fig — Heme analog uptake was evaluated by FI measurements from epimastigotes incubated for 5 minutes at 28°C with 100 μM of HAs (A). λex = 405 nm and λem = 583 nm for GaPP, 578 nm for ZnMP, 588 nm for ZnPP and 574 nm for SnMP. As control of treatment toxicity, the samples incubated for 5 minutes with these HAs were washed with PBS and suspended in LIT 10% FBS supplemented with 20 μM hemin. The cells were then maintained without periodic dilutions for 4 days and growth was monitored by cell counting in a Neubauer chamber (B). The results are representative of at least three independent experiments. The experimental data is presented as the mean ± SD. (TIF) [file pntd.0004359.s002.TIF]

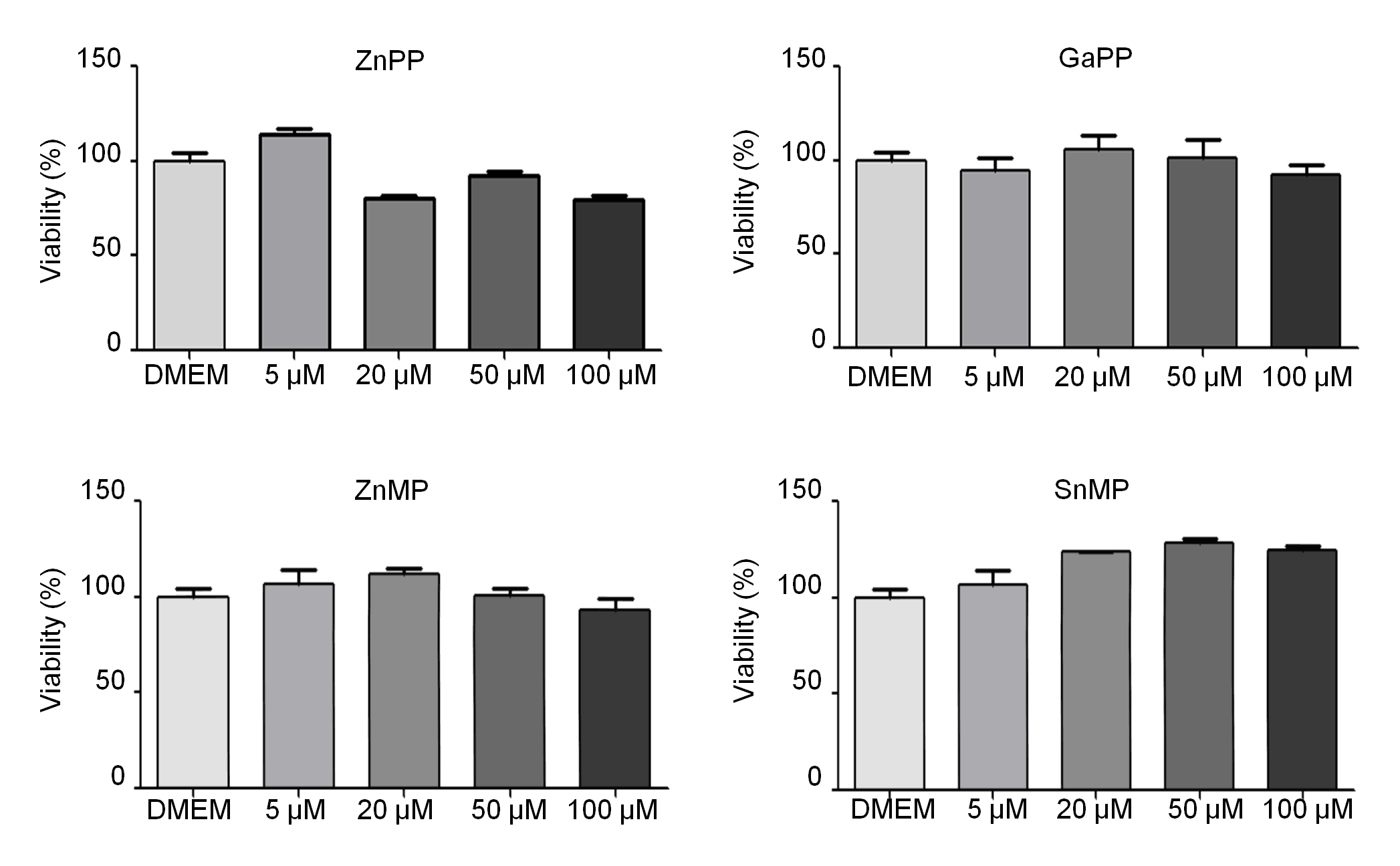

Supplement: S3 Fig — Vero cells were incubated for 2 hours at 37°C in a humid atmosphere containing 5% CO2, with different dilutions of the heme analogs and the cytotoxicity was measured with MTT viability assay. The results are representative of at least three independent experiments. The experimental data is presented as the mean ± SD. (TIF) [file pntd.0004359.s003.TIF]

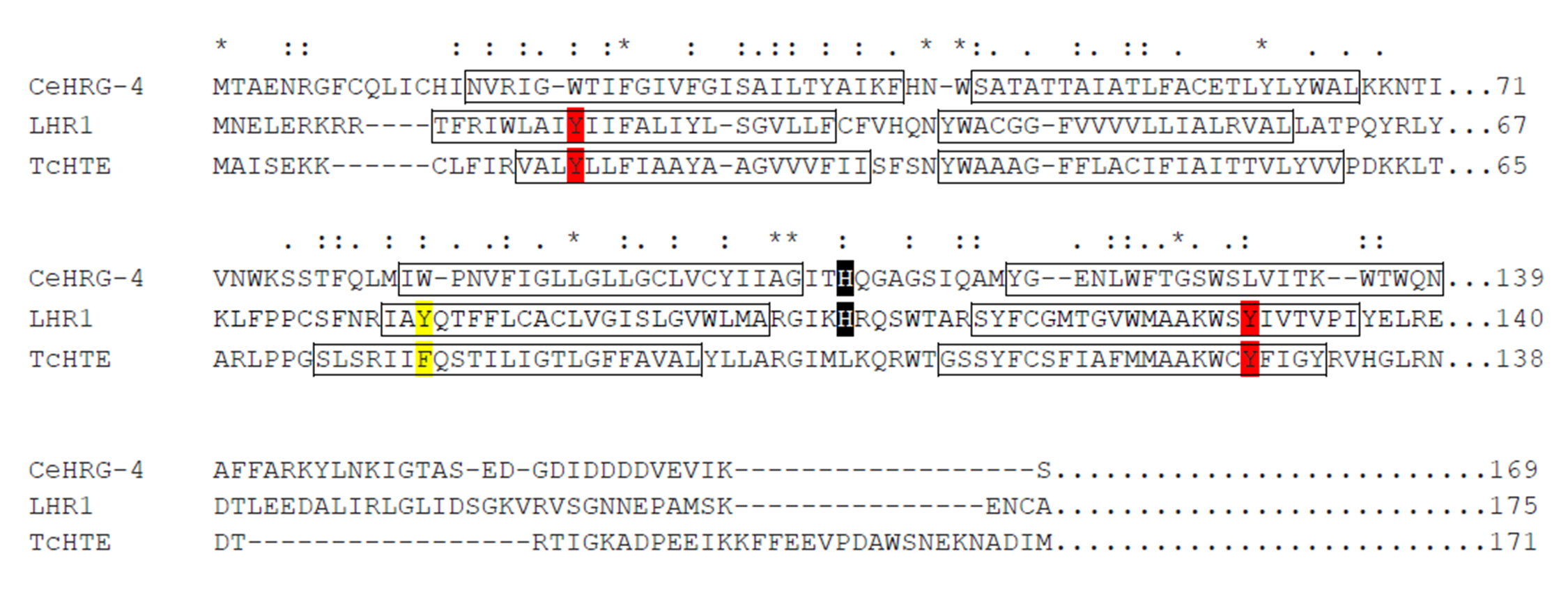

Supplement: S4 Fig — The alignment was performed using the Clustal W software [25], available at the EMBL-EBI website. Transmembrane-domains, represented by light frames, were predicted with TMHMM software [26], accessible from ExPASy Bionformatics Resource Portal. The reported conserved histidine residues of CeHRG-4 and LHR1 (residues H108 and H105 respectively) are outlined in black squares and according to these analyses, are absent in TcHTE. The Y18 reported as essential in LHR1 is conserved in TcHTE as well as Y129 (Y16, and Y127 in TcHTE) outlined in red, but Y80 which is not present, was replaced for F78 in TcHTE, outlined in yellow. (TIF) [file pntd.0004359.s004.TIF]

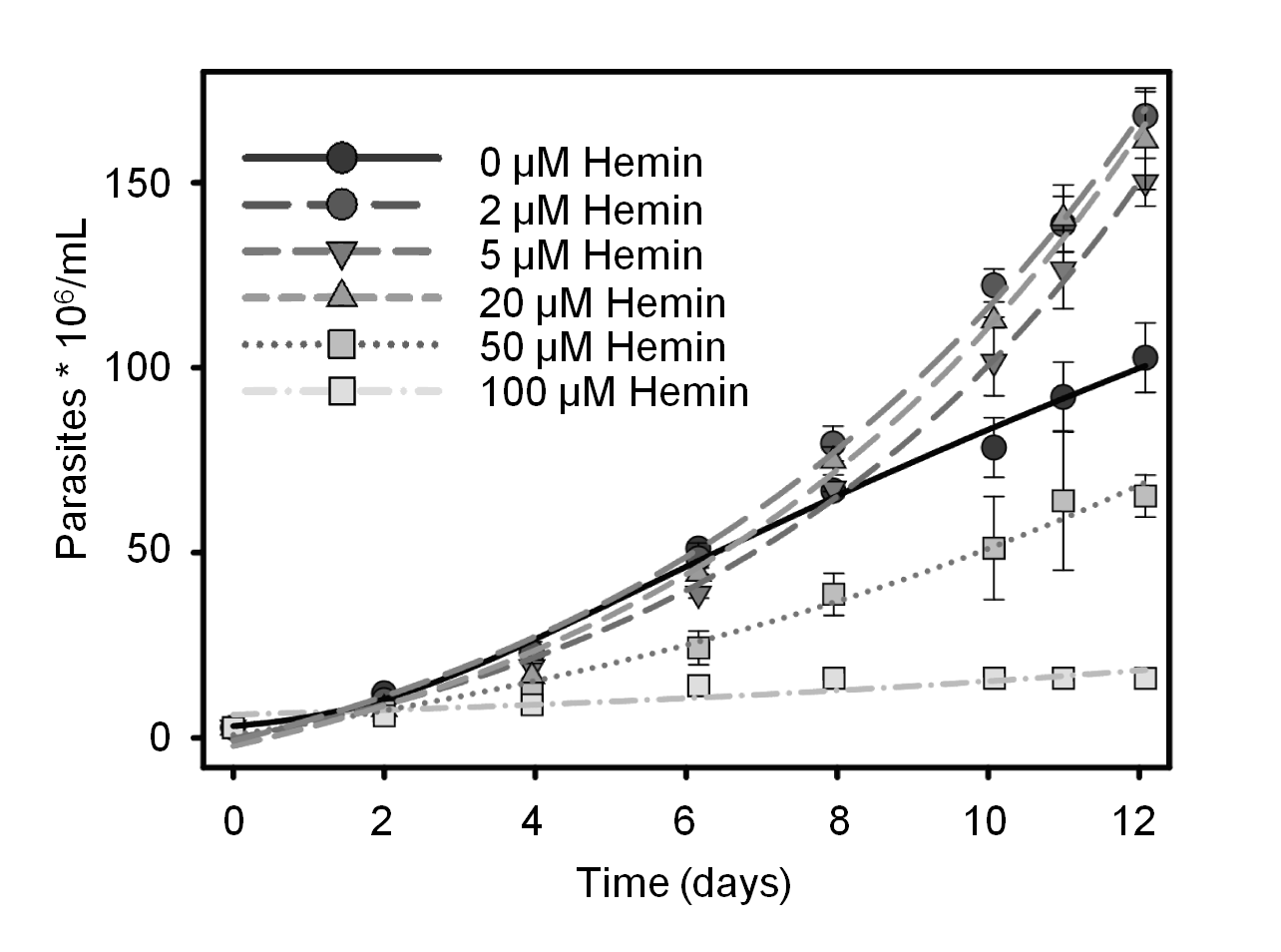

Supplement: S5 Fig — T. cruzi epimastigote maintained in LIT 10% FBS supplemented with 20 μM hemin in exponential growth phase were collected by centrifugation, washed twice with PBS and suspended at 4.5 x 10^6^ cells per 1.5 mL of LIT 10% FBS supplemented with 0, 2, 5, 20, 50 or 100 μM hemin. The cultures were maintained in the mid-log phase by periodic dilutions every 2 days and the growth was monitored by cell counting in a Neubauer chamber. The experimental data is presented as the mean ± SD. The results are representative of at least three independent experiments. (TIF) [file pntd.0004359.s005.TIF]
