# Supplementary material for: The Trypanosoma cruzi Protein TcHTE Is Critical for Heme Uptake
Source: PLoS Negl Trop Dis. 2016 Jan 11;10(1):e0004359. doi: 10.1371/journal.pntd.0004359 (PMC4713871; doi:10.1371/journal.pntd.0004359)
Supplement: S1 Text — (DOCX) [file pntd.0004359.s006.docx]

**Fluorescence intensity measurements of epimastigotes treated with HAs and control of treatment toxicity.**

The epimastigotes (20 x 10^6^ cells) previously incubated with 100 μM HAs were suspended with 500 μL of lysis buffer (0.5% Triton X-100 in PBS) and disrupted by repeated freezing and thawing cycles. The lysates were centrifuged and the supernatant (total cell-free extracts) was used to measure fluorescence intensity (FI) in a Varian Eclipse fluorometer, λex = 405 nm, recording the emission spectra between 450 to 650 nm, and analyzing the maximal emission at λem = 583 nm for GaPP, 578 nm for ZnMP, 588 nm for ZnPP and 574 nm for SnMP. To obtain the final value of IF, we subtracted the FI value obtained from a sample incubated 0 minutes with 100 μM of that the corresponding HA and normalized by parasite number. Data is expressed as means ± SD. The results are representative of at least three independent experiments.

As a control of the HAs treatment toxicity, the viability of the treated epimastigotes was tested. Briefly, samples of epimastigotes (20 x 10^6^ cells) incubated for 5 minutes with 100 μM HAs were washed three times with PBS and suspended in 3 mL of LIT 10% FBS supplemented with 20 μM hemin. The cells were then maintained without periodic dilutions for 4 days and growth was monitored by cell counting in a Neubauer chamber. The results are representative of at least three independent experiments.
